# Identification of an Epigenetically Marked Locus within the Sex Determination Region of Channel Catfish

**DOI:** 10.3390/ijms23105471

**Published:** 2022-05-13

**Authors:** Yujia Yang, Tao Zhou, Yang Liu, Changxu Tian, Lisui Bao, Wenwen Wang, Yu Zhang, Shikai Liu, Huitong Shi, Suxu Tan, Dongya Gao, Rex A. Dunham, Zhanjiang Liu

**Affiliations:** 1 The Fish Molecular Genetics and Biotechnology Laboratory, School of Fisheries, Aquaculture and Aquatic Sciences, Auburn University, Auburn, AL 36849, USA; yzy0042@auburn.edu (Y.Y.); zt@xmu.edu.cn (T.Z.); liuyang@dlou.edu.cn (Y.L.); tiancx@gdou.edu.cn (C.T.); baolisui@ouc.edu.cn (L.B.); wzw0029@auburn.edu (W.W.); yuzhang@ouc.edu.cn (Y.Z.); liushk@ouc.edu.cn (S.L.); huitong@stanford.edu (H.S.); szt0038@auburn.edu (S.T.); dunhara@auburn.edu (R.A.D.); 2 Fujian Key Laboratory of Genetics and Breeding of Marine Organisms, College of Ocean and Earth Sciences, Xiamen University, Xiamen 361102, China; 3 Department of Biology, College of Arts and Sciences, Syracuse University, Syracuse, NY 13244, USA; dgao09@syr.edu (D.G.); johnliu@syr.edu (Z.L.)

**Keywords:** fish, sex determination, methylation, transcriptome, epigenetics, RNA-Seq, sex differentiation

## Abstract

Channel catfish has an XY sex determination system. However, the X and Y chromosomes harbor an identical gene content of 950 genes each. In this study, we conducted comparative analyses of methylome and transcriptome of genetic males and genetic females before gonadal differentiation to provide insights into the mechanisms of sex determination. Differentially methylated CpG sites (DMCs) were predominantly identified on the sex chromosome, most notably within the sex determination region (SDR), although the overall methylation profiles across the entire genome were similar between genetic males and females. The drastic differences in methylation were located within the SDR at nucleotide position 14.0–20.3 Mb of the sex chromosome, making this region an epigenetically marked locus within the sex determination region. Most of the differentially methylated CpG sites were hypermethylated in females and hypomethylated in males, suggesting potential involvement of methylation modification in sex determination in channel catfish. Along with the differential methylation in the SDR, a number of differentially expressed genes within the SDR were also identified between genetic males and females, making them potential candidate genes for sex determination and differentiation in channel catfish.

## 1. Introduction

Sex determination is fascinating, not only because of its biological importance, but also because of its complexities and diverse mechanisms across a broad range of species in evolution. In most mammalian species and some insects, sex is determined by an XY sex determination system where males are heterogametic, whereas females are homogametic. In these species, sex is determined at the molecular level by the presence and expression of a dominant Y-linked gene that drives “maleness” (e.g., the SRY gene in mammals) [1,2]. In the XO sex determination system, females have two X chromosomes, whereas males have one X chromosome. Examples of XO sex determination have been demonstrated in grasshoppers [3], crickets [4], cockroaches [5], some mammals [6], *Caenorhabditis elegans* worms [7], and *Ancistrini ancistrus* [8], a catfish species within the order of Siluriformes. In insects, such as fruit flies [9], sex is determined by the ratio of X chromosome (X) to autosomes. In birds, some fish species, and insects [10,11,12], sex is determined by a ZW sex determination system, where the homogametic ZZ individuals are males, whereas the heterogametic ZW individuals are females. In ZW sex determination system, a W-linked dominant gene drives “femaleness”.

Teleost fish account for over half of all vertebrate species, having approximately 33,600 species [13]. They are also one of the most diverse groups of vertebrates in terms of sex determination [12]. To date, studies on sex determination have been conducted in only 270 fish species [14]. Of these, approximately 70% are male heterogametic (XY), and 30% are female heterogametic (ZW) [14]. At sub-chromosomal levels, both monogenic and polygenic sex determination systems have been demonstrated [15]. At the molecular level, various genes have been identified as the sex determination gene. For example, sdy in rainbow trout [16] and amhy in Patagonian pejerrey (*Odontesthes hatcheri*) [17] were identified as the master sex determination gene. Even in closely related medaka species, different genes were identified as the master sex determination gene, including dmy [18], dmrt1 [19], and gsdf [20], suggesting rapid evolution of sex determination mechanisms in teleosts. In addition to genetic factors, sex determination in teleost fish is also elastic and can be affected by environmental factors. With a given genetic composition, gonadal sex can be reversed by treatment with sex hormones or elevated temperature, stock density, pH condition, and hypoxia [21,22,23] in some fish species, suggesting the involvement of additional players in sex differentiation in addition to genetic sex determination.

Epigenetic factors have been reported to play crucial roles in sex determination, both in genetic sex determination (GSD) and environmental sex determination (ESD). In recent years, it has been reported that DNA methylation contributes to sex determination in plants [24,25,26,27,28], reptiles [29,30,31,32], and fish [29,30,32,33,34,35,36,37,38], although detailed mechanisms and extents of contribution vary. In melon, a transposon-induced DNA methylation modification in the promoter of CmWIP1 (a transcription factor) regulates the development of male, female, and hermaphroditic flowers [24]. In *Melandrium album*, a dioecious plant with an XY sex determination system, two X chromosomes in female plants showed differential methylation, and the Y chromosome in male plants was not hypermethylated [25]. In sea turtles, dimorphic DNA methylation profiles were found in bipotential and differentiated gonads during temperature-dependent sex determination [29]. Dimorphic DNA methylation patterns of certain genes, e.g., cyp19a1, were important for temperature-dependent sex determination in red-eared slider turtles and alligator embryos [30,32].

Among fish species, conserved patterns of DNA methylation leading to specific gene activation or silencing were observed in the developing gonads [39,40]. For example, the methylation and expression patterns of cyp19a1a and dmrt1 are both sex-specific and inversely correlated during gonadal development in Japanese flounder (*Paralichthys olivaceus*) [37,40]. In three-spine stickleback (*Gasterosteus aculeatus*), where sex is determined with an XY system [41,42], the majority of differentially methylated CpG sites (90%, 16,626 DMCs) showed a bias towards hypermethylation in females compared to males [38], and 65% of the differentially methylated sites were on chromosome 19, the sex chromosome in three-spine stickleback, suggesting the involvement of methylation in sex determination or sex differentiation processes for this species. Modifications in DNA methylation were also shown to contribute to ESD. Temperature-induced masculinization altered the global DNA methylation of many genes in the gonads of Nile tilapia (*Oreochromis niloticus*) [36]. In European sea bass, the altered methylation level of the promotor of gonadal aromatase cyp19a was shown to contribute to temperature-dependent sex determination [33]. In half-smooth tongue sole, the altered methylation in sex-reversed pseudomales was globally inherited by the offspring that could develop as pseudomales without incubation at a specific temperature [35].

**Figure 1:**
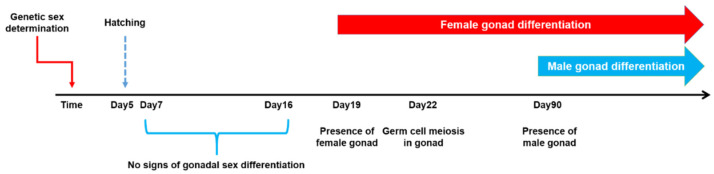
The time course of sex determination and gonadal differentiation in channel catfish, *Ictalurus punctatus*.

Channel catfish is a teleost fish species whose sex is genetically determined, but temperature can affect gonadal sex differentiation [43]. Treatment of fertilized eggs with high temperature or sex hormones can significantly affect sex ratios, leading to sex reversal from genetic males to phenotypic females [44,45]. Channel catfish has an XY sex determination system, but the sex chromosomes are cytologically indistinguishable [46]. Early studies excluded any roles of SRY, the sex determination gene in mammals, from being involved in sex determination in channel catfish [47]. Through genetic linkage mapping, chromosome 4 was identified as the sex chromosome [48], and the sex determination region (SDR) was mapped to a large physical distance due to the lack of recombination within the SDR [49]. Availability of the reference genomic sequence [50] and Y chromosome sequence [49] made it possible to conduct comparative sequence analysis. Identical gene content of 950 genes was found on both sex chromosomes [49], suggesting that sex of channel catfish is not determined by the presence of Y-specific gene(s) that are absent on X chromosome. Although genetic sex is determined at the time of fertilization, their gonadal feminization is not committed until at least 19 days post fertilization (dpf), whereas the testicular formation is not initiated until 90 dpf [22]. This differential timing of genetic sex determination and phenotypic sex differentiation offers a good model to study molecular mechanisms underlining sex determination and differentiation (Figure 1). In the present study, we determined sex-specific methylation and transcriptome profiles in genetic males (XY) and females (XX) of channel catfish during early gonadal differentiation. Here, we report an epigenetically marked locus within the SDR and the involvement of methylation and gene expression regulation in sex determination and differentiation of channel catfish.

## 2. Results

### 2.1. Genomic Landscape of Methylation

Whole-genome bisulfite sequencing was conducted using genetically female and male catfish samples at 9, 12, and 16 dpf. The bisulfite conversion rates of whole-genome bisulfite sequencing library construction were 99.50–99.76%. At 9 and 12 dpf, 77.1–78.4% of C’s in the CpG sites were methylated in both sexes. Some CpG sites were then demethylated, lowering the percentage of methylated CpG sites to 75.7 and 74.5% at 16 dpf in females and males, respectively (Table 1). In contrast to the high levels of CpG methylation, methylation of C within CHG and CHH sites (where H can be A, C, or T) was rare in both sexes, occurring only at levels of 0.3–0.4% (Table 1). The major methylated CHG types were C(A/T)G, and the major methylated CHH types were also A/T rich at both H sites (Supplemental Figure S1).

### 2.2. Differentially Methylated CpG Sites between Females and Males

**Figure 2:**
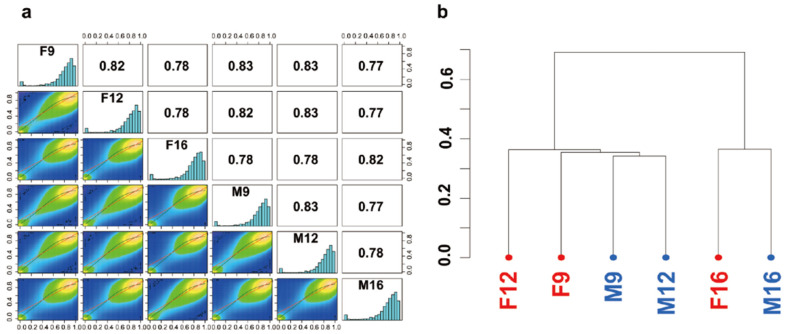
Correlation matrix and cluster analysis of methylation profiles in females and males of channel catfish, *I. punctatus*, at 9, 12, and 16 dpf. (**a**) The correlation matrix shows the Pearson correlation of genome-wide, base resolution CpG methylation between the female and male samples. Histograms showed the CpG methylation level from 0% to 100% using 20 bins (5% intervals). The scatter plots (lower left, colored half) showed the relationship of % methylation values for each sample pair using linear regression (red line) and loess fit (green line). The numbers in the grids (upper right half) showed the pair-wise Pearson’s correlation scores for each sample pair. (**b**) Clustering of female and male samples based on the similarity of methylation profiles.

A correlation matrix and hierarchical clustering analysis revealed subtle differences in whole-genome methylation profiles between the two sexes (Figure 2). The overall percentages of CpG methylation were similar between females and males. To identify the sexually differentially methylated CpG sites (hypermethylated and hypomethylated CpG sites), we conducted a differential methylation analysis using a cutoff of percent methylation difference greater than 25% and an FDR-corrected *p*-value smaller than 0.05. Based on these criteria, a small fraction of the methylated CpG sites were differentially methylated, 156 and 247 at 9 and 12 dpf, respectively, between genetic males and females. At 16 dpf, differentially methylated CpG sites between the sexes increased dramatically to 2261 (Supplemental Table S1). Females had more hypermethylated CpG sites than males. There were 101, 166, and 1310 hypermethylated CpG sites in females, compared to 55, 81, and 951 hypermethylated CpG sites in males, at 9, 12, and 16 dpf, respectively (Table 2).

To determine the distribution of differentially methylated CpG sites in the genome, the CpG sites were mapped to the reference genome sequence [50]. The sex chromosome, chromosome 4, contained much higher density of differentially methylated CpG sites (Table 2). Of the 29 channel catfish chromosomes, the sex chromosome harbored 51.3%, 34.4%, and 42.9% of all differentially methylated CpG sites at 9 dpf, 12 dpf, and 16 dpf, respectively (Table 2). If the distribution was even across all the chromosomes, chromosome 4 was expected to harbor 4.5% of differentially methylated CpG sites according to its size, suggesting that differential methylation may be associated with the sex determination process.

### 2.3. Differentially Methylated CpG Sites between Females and Males

**Figure 3:**
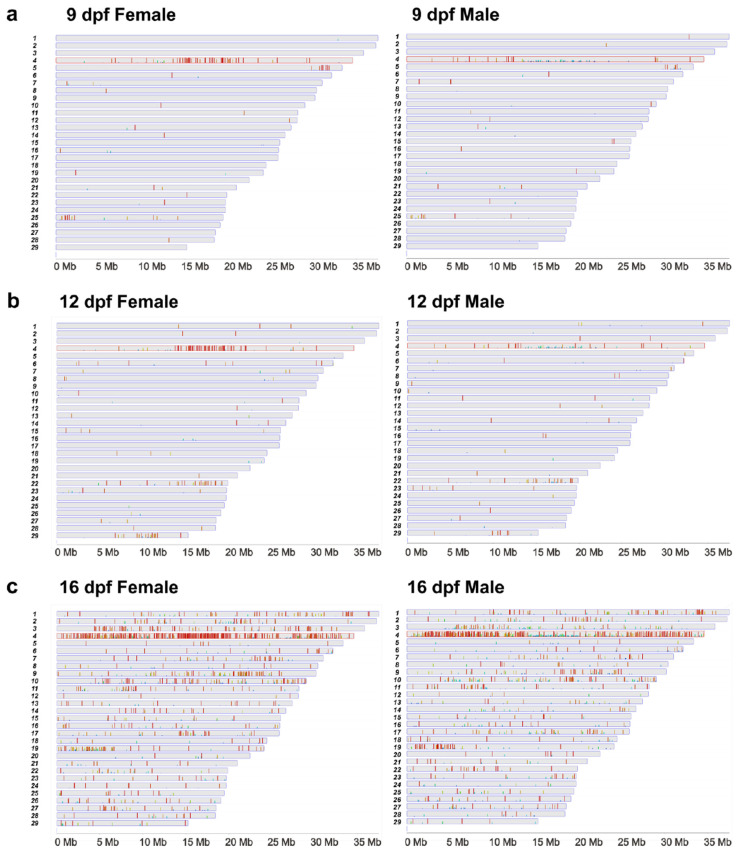
Differentially methylated CpG sites in the two sexes of channel catfish, *I. punctatus* at 9 dpf (**a**), 12 dpf (**b**), and 16 dpf (**c**). Red color indicated hypermethylation while blue color indicated hypomethylation, relative to each other of genetic females and males.

The sex chromosome harbored significantly more differentially methylated CpG sites, and the SDR also had a much greater density of differentially methylated CpG sites compared to the rest of the sex chromosome (Figure 3). Specifically, chromosome 4 had 80 differentially methylated CpG sites (59 hyper- in females and 21 hyper- in males) at 9 dpf, and 47 of them were in the SDR. Similarly, at 12 dpf, 85 differentially methylated CpG sites (64 hyper- in females and 21 hyper- in males) were found on the sex chromosome, of which 60 were found in the SDR (Table 2). At 16 dpf, the overall differentially methylated CpG sites increased dramatically, making the proportion of differentially methylated CpG sites on the sex chromosome and those in the SDR less distinctive than at 9 and 12 dpf, but the density of differentially methylated sites on the sex chromosome was still significantly higher than on other chromosomes, with 971 differentially methylated CpG sites (569 hyper- in females and 402 hyper- in males) on the sex chromosome out of 2261 in the whole genome. The differentially methylated CpG sites outside of the SDR also increased drastically at 16 dpf, making the density of differentially methylated CpG sites less distinctive in the SDR, with 316 within SDR, out of the 971 differentially methylated CpG sites on the sex chromosome.

**Figure 4:**
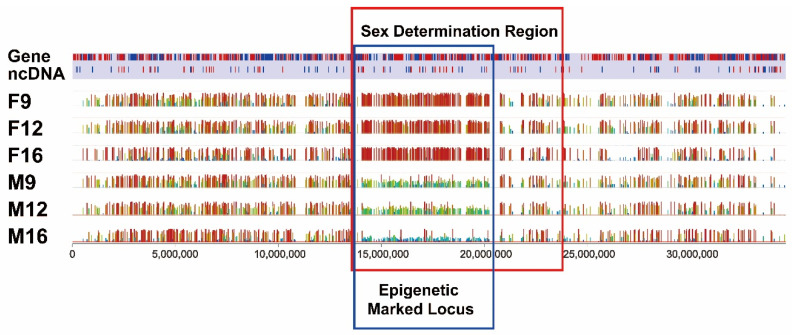
The epigenetically marked locus (bordered by a blue box) within the SDR (bordered by a red box) in channel catfish, *I. punctatus*, with the red color representing hypermethylation, green color representing medium methylation, and blue color representing hypomethylation.

Not only the numbers of differentially methylated sites were most dense within the SDR of chromosome 4, but also the levels of methylation differences were most striking within the SDR. The most differentially methylated region was within the SDR but a smaller region, approximately 14.0–20.3 Mb from the start of the sex chromosome (Figure 4), making this chromosome region epigenetically marked. Within this region, the CpG sites were hypermethylated in females (Figure 4). In contrast, the CpG sites within this region were hypomethylated in males (Figure 4). Taken together, an epigenetically marked locus was well defined (14.0–20.3 Mb) within the SDR with drastically different patterns of methylation between genetic males and genetic females.

### 2.4. Identification of Differentially Methylated Genes within SDR

A total of 96, 153, and 1120 differentially methylated genes (DMGs) were identified in the genome at 9, 12, and 16 dpf, and a significant fraction, 43, 50, and 308 DMGs, were found on chr4 (Table 3). Within the SDR, 23, 31, and 77 genes were observed to be differentially methylated between sexes at 9, 12, and 16 dpf (Table 3, Supplemental Table S2). Of hypermethylated genes identified in females at 9, 12, and 16 dpf, ten genes were common to all stages, and they were all located within the SDR (Supplemental Figure S2). These ten genes were: polr2a, idh2, rasgrf1, chd2, nectin1, sema4b, vasp, rasa2, zbtb38, and trip12. There were considerably fewer hypermethylated genes in males at 9, 12, and 16 dpf, of which only one was common to all stages. This gene was cdh13 (cadherin gene), and it was located outside of the SDR between 9,328,577 bp and 9,747,822 bp.

### 2.5. Differentially Expressed Genes within the SDR

RNA-Seq analyses were conducted to identify differentially expressed genes between genetic females and males during early sex differentiation. At 7, 12, and 17 dpf (days post fertilization), 46, 112, and 300 genes were differentially expressed between genetic females and males, respectively (Supplemental Tables S3 and S4). We focused further analysis on the sex determination region, where a total of 8 genes were differentially expressed at least at one time point. Of the 8 DEGs within the SDR, six genes (hydin-1, LOC108264027, gtf2ird2b, kcnj1, LOC108264687, actrt3) were expressed at higher levels in genetic males, and two genes (cipc, LOC108264260) were expressed at higher levels in genetic females, with a cutoff threshold of fold change larger than 2 and Q-value (FDR-adjusted *p*-value) smaller than 0.05 (Table 4). Three genes were differentially expressed at higher levels as early as 7 dpf, including hydin-1, an uncharacterized gene LOC108264027, and gtf2ird2b. The other three DEGs with higher expression in genetic males were kcnj1, LOC108264687, and actrt3, and their differential expression was not detected until 17 dpf (Table 4). Two DEGs (cipc and LOC108264260) with higher expression in genetic females were detected at 7 dpf or later (Table 4). Of the 8 DEGs, hydin-1 gene was differentially expressed at all time points, and also most dramatic, ranging from 8.96 to 41.56 times higher in genetic males than in genetic females (Table 4). The X allele of hydin-1 was observed with very low expression with patterns of hypermethylation as compared to its Y allele (unpublished data).

Within the SDR, three genes (LOC108264027, gtf2ird2b, and cipc) were differentially methylated between genetic females and males, which were observed with hypermethylation in females and hypomethylation in males. On chromosome 4, five additional genes were also both DMGs and DEGs, including slc12a3, lctl, asz1, si, and LOC108263970 (Supplemental Table S5). Ten genes on the other chromosomes were both DMGs and DEGs, including cpb1 (chr1), frem3 (chr3), kcnk12 (chr3), upp2 (chr6), plb1 (chr9), LOC108273626 (chr13), spg20 (chr16), mmp13 (chr16), cfap77 (chr22), and ptprh (chr23) (Supplemental Table S5).

## 3. Discussion

Channel catfish has cytologically indistinguishable X and Y chromosomes through karyotype analysis [46], and high similarities were observed in sequence identity and gene contents of X and Y chromosomes [49]. Through genetic linkage mapping and GWAS analyses, chromosome 4 was identified as the sex chromosome, and the sex determination region was identified with 621 significant sex-associated SNPs identified by GWAS [49]. However, whether the epigenetic modification is involved in sex determination of channel catfish remains to be revealed. In this study, we determined genome-wide DNA methylation with single-base resolution in genetic females and males of channel catfish. Our results showed that DNA methylation is frequent in the catfish genome at CpG sites, with 74.5–78.4% of all C’s at the CpG sites being methylated. This is similar to those observed in other animal species, slightly higher than that in mice (74%) [51] and tilapia (69.60%) [52], but slightly lower than that in zebrafish (80.3%) [51]. Methylation rate at CHG and CHH sites in channel catfish was very low at 0.3–0.4%, lower than that seen in zebrafish (1.22% and 0.91%, respectively) [51], but comparable to that observed in tilapia (0.47% and 0.57%) [52], and that of tiger pufferfish (0.3% and 0.9%) [53]. In contrast to the low methylation rates at CHG and CHH sites in fish, high levels of methylation at CHG and CHH sites have been widely observed in plants, where such methylation regulates gene expression [54].

After determination of differentially methylated CpG sites between genetic females and males, high density of sexually differentially methylated CpG sites were observed on the sex chromosome (chromosome 4), especially within the SDR. Within the SDR, the majority of differentially methylated CpG sites were hypermethylated in females. In contrast, the SDR was hypomethylated in males, making this an epigenetically marked locus (EML). The EML alignment with the SDR suggested the involvement of epigenetic modification in sex determination of channel catfish. This result was consistent with a recent methylation study in another fish species, threespine stickleback, which also has an XY sex system [38], where a higher proportion of differentially methylated CpG sites was observed on sex chromosome chr19 [38].

Within the SDR, many genes were observed to be differentially methylated between sexes. Among them, several differentially methylated genes within the SDR were also found to be located in the sex determination locus of other species. For instance, idh-rasgrf1-sema4b are three known genes in the master sex determination locus on LG19 in three-spine stickleback [42]. The idh2 loci were tightly linked to the sex determination element in conchostracan shrimp, *Eulimnadia texana* [55]. Chd2 is one of the sex chromosome-linked genes used for sex identification in birds [56,57]. Other genes within the SDR of channel catfish functionally related to sex differentiation and gonadal development in other species included a cell adhesion gene, nectin1, which was explicitly upregulated in the sexually differentiating gonads of mice [58], and E3 ubiquitin-protein ligase trip12 gene, which play a role in chromatin modification of early germ cells in rat testis [59]. It is possible that many genes involved in sex determination and differentiation are simply co-localized in the genome as functional hubs [60].

We also conducted transcriptome analyses of genetic females and males during the critical period of sex differentiation. We then analyzed the correlation between gene body/promotor DNA methylation and gene expression (Supplemental Figure S3), but no clear positive or negative correlation was found in the present study. The relationship between DNA methylation and gene expression was found to be complicated, depending on the position of methylation in relation to the methylated gene parts, e.g., promoters, exons, and introns, among other factors. Future studies are warranted to determine the relationship between methylation and gene expression.

Within the SDR, a total of eight genes were differentially expressed between males and females at three tested time points. Hydin-1 was the most significant male biased gene within the SDR; its expression levels were 8.96–41.56 times higher in males than in females. Hydin is a structural protein within the axoneme of sperm flagellum required for ciliary motility [61]. In our previous study of male channel catfish, hydin gene was observed with male-biased expression in the testis transcriptome [62]. In zebrafish, abnormal downregulated expression of hydin gene was observed in androgen signaling-depleted individuals with defective testicular organization and spermatogenesis [63]. In a similar situation as hydin-1, but to a lesser extent, the other five genes in SDR that were expressed higher in males than in females, including LOC108264027, gtf2ird2b, kcnj1, LOC108264687, and actrt3. Expression levels of LOC108264027 were over two times higher in males at 7, and 12 dpf, and it was also sexually differentially methylated. A general transcription factor Gtf2ird2b was differentially expressed, with over two times higher expression in males than in females, and the methylation levels were higher in females than in males as well. Gtf2ird2b was located within sex-related QTL in red-tail catfish *Hemibagrus wyckioides* [64], whose expression pattern between females and males and potential roles in sexual differentiation has not been reported.

Two genes were expressed at significantly higher levels in females, and they were, cipc and LOC108264260. Cipc is a potential negative-feedback regulator in circadian-clock mechanisms that control daily rhythms of physiology and behavior [65]. Cipc was differentially expressed at 7, 12, and 17 dpf. Cipc was hypermethylated in females and hypomethylated in males. In *Takifugu rubripes*, estrodiol-17β-induced feminization significantly depressed the gene expression of cipc [66]. The differential expression patterns during the critical period of sex differentiation suggested the involvement of gene expression within SDR for sex determination in channel catfish.

In addition to genes within SDR, DMGs and DEGs were also identified outside of SDR on chr4 or from other chromosomes. On chr4, asz1 showed higher expression levels in male catfish, which was hypermethylated in females but hypomethylated in males. The homolog of asz1 in mice plays an essential structural role in male meiosis and silencing the retrotransposon expression in the male germline [67]. In addition to the sex chromosome, cpb1 on chr1 was highly expressed in male catfish and also differentially methylated between genetic females and males, whose homolog in *Drosophila* was associated with regulating spermatogenesis and sperm cell fate [68]. Even though there are no previous reports of the roles of many of these genes in sex differentiation or gonadal development, future studies of their potential roles in sex differentiation in channel catfish are warranted.

The identification of the epigenetically marked locus within the SDR, and its related differential expression of genes within the SDR suggested epigenetic control of sex determination in channel catfish. While epigenetic mechanisms have been reported to exert crucial roles in the process of both genetic and environmental sex determination of a number of fish species, for example, gonadal sex differentiation is highly elastic such that environmental cues, exogenous exposure to sex hormones, or perturbation of expression of many genes related to sex differentiation may cause sex reversal [35,40,69]. In the present study, we identified sex-specific differential methylation and differential expression within the SDR in channel catfish. High density of sexually differentially methylated CpG sites were located within SDR, suggesting potential epigenetic regulation of sex determination or gonadal differentiation in channel catfish. The genes that are differentially methylated and differentially expressed before the commitment of sex phenotypes between genetic males and genetic females might be candidates for sex determination and differentiation gene(s) in channel catfish. Studies of the inheritance of epigenetic marks and gene functional analyses are warranted in the future.

## 4. Materials and Methods

### 4.1. Experimental Fish

Experimental protocols for animal care and tissue collection were approved by the Auburn University Institutional Animal Care and Use Committee (AU-IACUC). Broodstock preparation, artificial spawning, fertilization, and husbandry procedures were performed according to previously published protocols [70]. In this study, egg batches were incubated at 28 °C. In a previous study, channel catfish would produce balanced sex ratios at this temperature [22,43]. Hatching occurred approximately 5 dpf.

### 4.2. Tissue Collection and DNA Isolation

Channel catfish fry were euthanized and individually sampled prior to sexual differentiation at 9, 12, and 16 dpf with MS-222 (200 mg/L, Syndel Laboratories, Vancouver, BC, Canada). The genetic sex of each individual was determined using a catfish sex-linked microsatellite marker (forward primer: 5′-ACATCGCTTTGAGAAGCTGC-3′; reverse primer: 5′-GTGAATGTGAGACTAACAGGAGG-3′) [71]. Genomic DNA extracted from the head and tail portions were used for genetic sex identification using PCR. Genomic DNA extracted from larvae was used for whole genome bisulfite sequencing. DNA was extracted using a DNeasy Blood and Tissue Kit (Qiagen, Valencia, CA, USA) following the manufacturer’s protocol. For genotypic female or male catfish, an equal amount of genomic DNA was pooled together from five fish for each genetic sex. RNase A (17,500U, Qiagen, Valencia, CA, USA) was used to generate RNA-free gDNA for bisulfite conversion and whole-genome bisulfite sequencing (WGBS).

### 4.3. Bisulfite Conversion, Library Preparation, and Sequencing

After testing the quality of the DNA samples, lambda DNA was added as a negative control. Bisulfite treatment of genomic DNA was performed using the EZ DNA Methylation Gold Kit (Zymo Research, Orange, CA, USA). Library construction and WGBS with 30X coverage were performed as previously described [72]. The libraries were sequenced with a HiSeq X instrument (Illumina, San Diego, CA, USA).

### 4.4. Alignment of Reads and Calculation of the Methylation Level

Raw reads were filtered for base quality (≥15) and read length (≥36) using Trimmomatic [73]. The clean reads were aligned against the channel catfish reference genome assembly [50] using Bismark [74], and methylation calls (CpG, CHH, and CHG sites) were extracted using the Bismark methylation extractor [74]. The output coverage files were used for the analysis and graphical visualization of the differential methylation calculations.

### 4.5. Differential Methylation Analysis

Cytosine methylation levels at single base-pair resolution were calculated and visualized using Seqmonk (Babraham Bioinformatics, Cambridge, UK). To identify the hypermethylated (high methylation level) and hypomethylated (low methylation level) CpG sites between females and males, the differential methylation analysis was conducted using the chi-squared test in Seqmonk with a cutoff point of percent methylation difference greater than 25% and an FDR-corrected *p*-value < 0.05. A minimum read coverage of 10× was set as a minimum parameter for calling methylation states. The CpG methylation clustering and Pearson correlation plots were generated using MethylKit [75]. Non-CG methylation sequence (CHH and CHG) motifs and logos were built by custom python scripts and ggseqlogo in R [76].

### 4.6. High-Throughput RNA Sequencing

The fry at 7, 12, and 17 dpf were euthanized with MS-222 (200 mg/L, Syndel Laboratories, Vancouver, BC, Canada) before sexual differentiation occurred and were sampled individually. The genetic sex of each individual was identified using a catfish sex-linked marker [71]. Total RNA was isolated from the genotypic female and male catfish using the RNeasy Plus Universal Kit (Qiagen, Valencia, CA, USA) according to the manufacturer’s instructions. Each sample has three replicates, and each replicate has equal amounts of RNA from three individuals of each sex. The RNA-Seq libraries were prepared following the standard protocols and were sequenced on an Illumina HiSeq X platform.

### 4.7. Identification of Sexually Dimorphic Expressed Genes

The low-quality reads (average base quality lower than 15), short reads (length smaller than 36), and adapters in the raw RNA-Seq reads were trimmed using Trimmomatic [73]. The trimmed high-quality reads were mapped to the channel catfish genome [50] using TopHat [77]. The aligned RNA-Seq reads and channel catfish annotation files were input into the Cufflinks pipeline [77] to detect the differentially expressed genes. At each time point (7, 12, and 17 dpf), the expression value (fragments per kilobase of transcript per Million mapped reads, FPKM) of genes from female and male samples were compared. The statistical significance was calculated based on whether the *p*-value was higher than the Q-value (FDR-adjusted *p*-value).

## Figures and Tables

**Table 1:** Summary of methylated CpG, CHG, CHH sites and their percentages in whole genome methylation profiles of male and female channel catfish, *I. punctatus*, during the critical period of sex determination.

Groups	Methylated CpG	Methylated CHG	Methylated CHH
**Female**			
9 dpf	258,326,088 (77.1%)	2,817,165 (0.4%)	7,706,815 (0.3%)
12 dpf	217,250,994 (77.2%)	2,156,312 (0.3%)	6,284,900 (0.3%)
16 dpf	284,031,704 (75.7%)	2,814,003 (0.3%)	8,120,484 (0.3%)
**Male**			
9 dpf	253,621,156 (77.5%)	2,718,707 (0.4%)	7,540,415 (0.3%)
12 dpf	277,521,207 (78.4%)	3,124,962 (0.4%)	8,583,702 (0.4%)
16 dpf	290,874,272 (74.5%)	2,922,346 (0.3%)	8,239,077 (0.3%)

**Table 2:** Summary of methylated CpG sites in the genome of male and female channel catfish, *I. punctatus*, during the critical period of sex determination. Chromosome 4, the sex chromosome, is highlighted.

Chr	9 dpf		12 dpf		16dpf	
	♀ Hyper	♂ Hyper	♀ Hyper	♂ Hyper	♀ Hyper	♂ Hyper
1	0	1	3	3	62	63
2	0	1	2	2	32	26
3	0	0	0	0	81	52
4	59	21	64	21	569	402
5	9	6	0	1	9	4
6	1	1	9	3	16	11
7	2	3	4	1	28	18
8	1	0	3	2	10	11
9	0	0	0	1	90	39
10	1	1	1	1	70	64
11	1	1	1	2	30	25
12	1	1	1	2	6	4
13	1	1	2	0	18	11
14	1	0	2	2	19	9
15	0	2	4	0	12	11
16	2	1	1	2	20	12
17	0	0	0	0	31	38
18	0	0	4	1	6	10
19	1	2	2	1	80	48
20	0	0	0	0	3	8
21	2	4	1	1	7	9
22	1	0	28	18	12	17
23	1	1	4	6	10	10
24	0	0	0	1	9	4
25	16	8	0	1	19	10
26	0	0	1	1	18	12
27	0	0	2	1	22	15
28	1	0	2	0	12	4
29	0	0	25	7	9	4
**Total**	**101**	**55**	**166**	**81**	**1310**	**951**

**Table 3:** Numbers of differentially methylated CpG sites and differentially methylated genes between genetic male and female fish in the whole genome and on chromosome 4 of channel catfish, *I. punctatus*.

Stage	Differentially Methylated CpG Sites			Differentially Methylated Genes		
	Whole Genome	Chr4	SDR	Whole Genome	Chr4	SDR
9 dpf	156	80	47	96	43	23
12 dpf	247	85	60	153	50	31
16 dpf	2261	971	316	1120	308	77

**Table 4:** Differentially expressed genes within the sex determination region between genetic females and males of channel catfish, *I. punctatus*. Positive and negative numbers indicate higher expression in genetic males and genetic females, respectively, while “nd” indicates no difference between genetic females and males.

Gene	Differential Expression FPKM Male/FPKM Female			Differentially Methylation		
	7 d	12 d	17 d	9 d	12 d	16 d
hydin-1	8.96	15.05	41.56	nd	X Hyper	X Hyper
LOC108264027	2.08	2.29	nd	nd	♀ Hyper	♀ Hyper
gtf2ird2b	2.83	2.35	2.70	nd	♀ Hyper	♀ Hyper
kcnj1	nd	nd	2.75	nd	nd	nd
LOC108264687	nd	nd	2.24	nd	nd	nd
actrt3	nd	nd	2.07	nd	nd	nd
cipc	−2.18	−2.78	−2.61	nd	♀ Hyper	♀ Hyper
LOC108264260	nd	nd	−2.29	nd	nd	nd

## Data Availability

Sequence data have been submitted have been deposited at the NCBI BioProject database under accession PRJNA553256.

## Supplementary Materials

The following supporting information can be downloaded at: https://www.mdpi.com/1422-0067/23/10/5471/s1.
