# Supplementary material for: Identification of an Epigenetically Marked Locus within the Sex Determination Region of Channel Catfish
Source: Int J Mol Sci. 2022 May 13;23(10):5471. doi: 10.3390/ijms23105471 (PMC9171582; doi:10.3390/ijms23105471)
Supplement: Supplementary file 1 [file ijms-23-05471-s001.zip › ijms-1696349-supplementary/Supplementary Files/Supplemental Files.pdf]

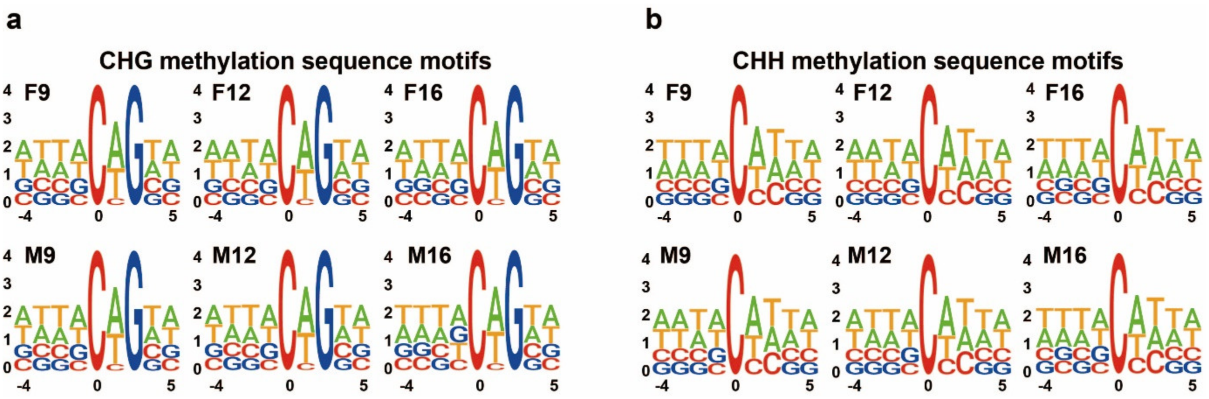

Supplementary Figure S1. The methylation sequence motifs of CHG and CHH in two sexes of channel catfish at 9, 12, and 16 dpf.

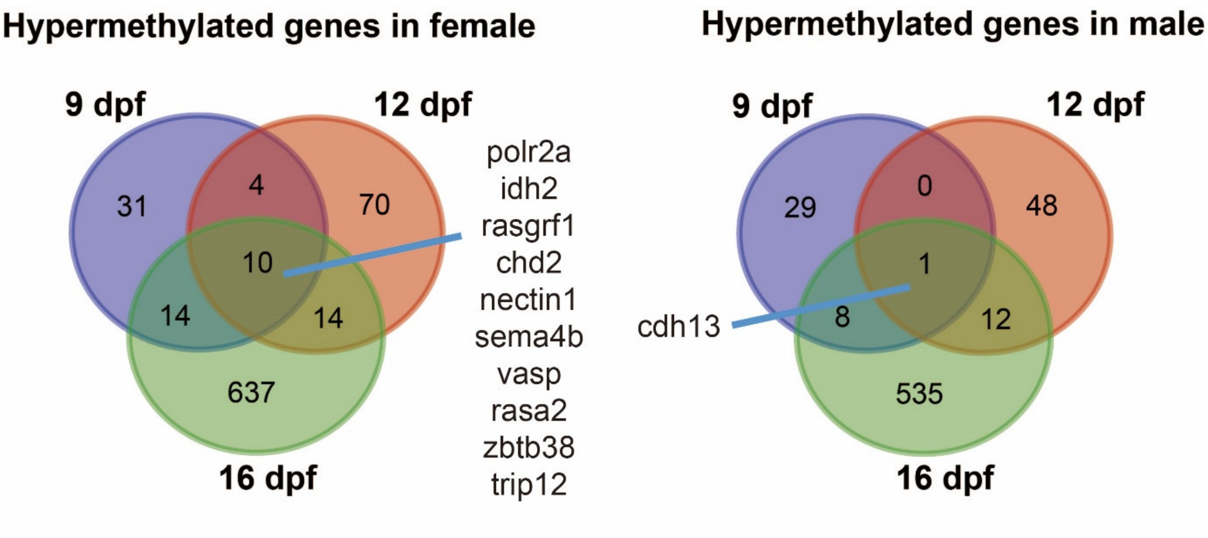

Supplementary Figure S2. The number of hypermethylated genes in females or males at 9, 12, and 16 dpf in females and males. 10 genes (polr2a, idh2, rasgrf1, chd2, nectin1, sema4b, vasp, rasa2, zbtb38, and trip12) were hypermethylated in females at all three timepoints of 9, 12, and 16 dpf, and one gene, cdh13, was hypermethylated in males at all three timepoints of 9, 12, and 16 dpf.

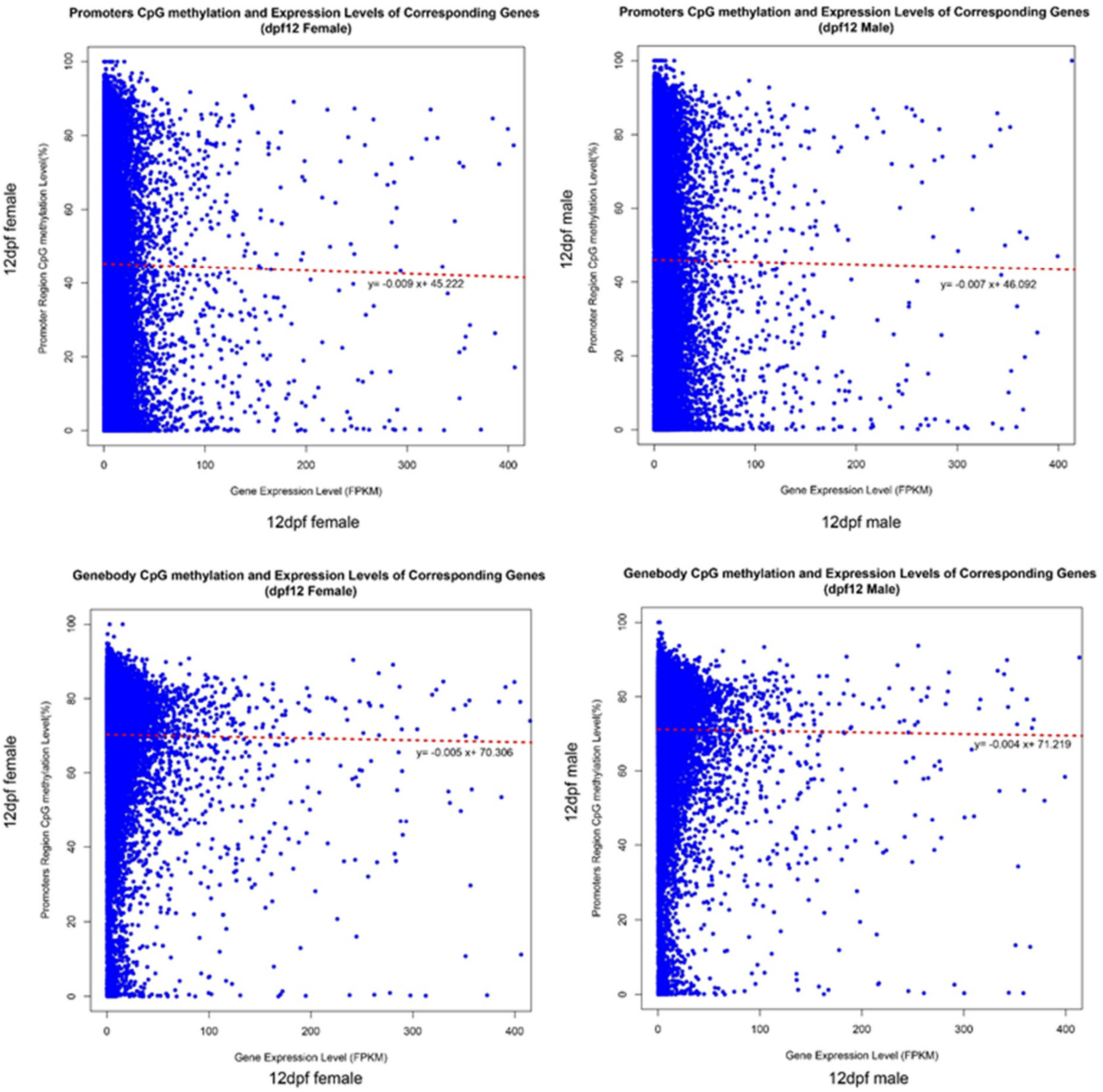

Supplementary Figure S3. Correlation between CpG methylation levels (promoter and gene body) and respective gene expression levels in channel catfish.

Supplementary Table S2. Differentially methylated genes between genetic females and males in the sex determination region of channel catfish, *Ictalurus punctatus*. Blue shade "+" represents that the gene was differentially methylated between genetic females and males.

Gene	9 dpf	12 dpf	16 dpf
polr2a	+	+	+
idh2	+	+	+
rasgrf1	+	+	+
chd2	+	+	+
nectin1	+	+	+
sema4b	+	+	+
vasp	+	+	+
rasa2	+	+	+
zbtb38	+	+	+
trip12	+	+	+
LOC108264027	+	+	
LOC108264215	+	+	
shank2	+	+	
cdon	+		+
sptbn4	+		+
pik3c2a	+		+
sphkap	+		+
pdcd10	+		+
LOC108264720	+		+
prdm10	+		+
ddx6		+	+
LOC108263952		+	+
gtf2ird2b		+	+
LOC108264255		+	+
pid1		+	+
sertad3		+	+
reln		+	+
LOC108264017		+	+
gnao1		+	+
cipc		+	+
dhx34		+	+
sec11a	+		
LOC108264030	+		
esrrg	+		
LOC108263945		+	
shkbp1		+	
LOC108264264		+	
LOC108264015		+	
LOC108264022		+	
st14		+	
hydin-l2		+	
slc28a2			+

LOC108264806			+
LOC108264221			+
hsf4			+
prx			+
LOC108264443			+
fan1			+
tmem170a			+
aplp2			+
LOC108264200			+
fam65a			+
nrn1l			+
dner			+
spred1			+
carmil2			+
akt2			+
blm			+
lmo2			+
ranbp10			+
bcl9l			+
pard6a			+
treh			+
araf			+
LOC108264345			+
cfdp1			+
napa			+
slco3a1			+
daw1			+
LOC108263932			+
LOC108264718			+
mef2a			+
LOC108264019			+
cbfb			+
nlrc3			+
irf2bp1			+
bcar1			+
pskh1			+
necab2			+
LOC108264797			+
nutf2			+
LOC108264373			+
mbtps1			+
polr2i			+
golim4			+
srpra			+
ppfia1			+
ctcf			+

pik3c2a			+
st14			+
**Total**	**23**	**31**	**77**

Supplementary Table S4. Distribution of differentially expressed genes between genetic females and males at 7, 12, and 17 dpf across the 29 chromosomes of channel catfish, *Ictalurus punctatus*.

**Chromosome**	**Number of differentially expressed genes**		
	**7dpf**	**12dpf**	**17dpf**
Chr 1	4	5	17
Chr 2	1	1	14
Chr 3	1	4	14
Chr 4	8	7	14
Chr 5	0	5	14
Chr 6	1	6	12
Chr 7	3	4	8
Chr 8	1	1	9
Chr 9	0	2	10
Chr 10	1	8	8
Chr 11	0	6	6
Chr 12	4	8	13
Chr 13	0	7	13
Chr 14	1	2	10
Chr 15	1	7	8
Chr 16	3	2	13
Chr 17	0	4	12
Chr 18	1	4	17
Chr 19	1	0	7
Chr 20	1	6	9
Chr 21	3	8	6
Chr 22	1	1	7
Chr 23	0	5	6
Chr 24	2	0	8
Chr 25	1	1	9
Chr 26	4	3	12
Chr 27	2	2	6
Chr 28	1	0	5
Chr 29	0	3	13
**Total**	**46**	**112**	**300**

Supplementary Table S5. Differentially expressed and methylated genes outside of SDR region during critical period of sex determination in channel catfish.

Gene	chr	Differential expression FPKM male / FPKM female			Differentially methylation Female / Male		
		7d	12d	17d	9d	12d	16d
slc12a3	chr4	nd	nd	2.90	nd	♂ Hyper	♂ Hyper
lctl	chr4	nd	nd	2.17	nd	♂ Hyper	nd
asz1	chr4	nd	nd	2.04	nd	nd	♀ Hyper
si	chr4	nd	-2.72	nd	nd	nd	♂ Hyper
LOC108263970	chr4	nd	-4.01	-2.65	nd	nd	♂ Hyper
cpb1	chr1	nd	nd	2.92	nd	nd	♂ Hyper
frem3	chr3	nd	nd	2.09	nd	nd	♀ Hyper
kcnk12	chr3	nd	nd	-2.67	nd	nd	♀ Hyper
upp2	chr6	nd	nd	-2.13	nd	♀ Hyper	nd
plb1	chr9	nd	-2.27	nd	nd	nd	♀ Hyper
LOC108273626	chr13	nd	nd	2.01	nd	nd	♀ Hyper
spg20	chr16	nd	nd	2.04	nd	nd	♂ Hyper
mmp13	chr16	-2.08	nd	nd	nd	nd	♂ Hyper
cfap77	chr22	nd	nd	2.06	nd	nd	♀ Hyper
ptprh	chr23	nd	-2.05	nd	♀ Hyper	nd	nd
